# STK33 participates to HSP90-supported angiogenic program in hypoxic tumors by regulating HIF-1α/VEGF signaling pathway

**DOI:** 10.18632/oncotarget.20535

**Published:** 2017-08-24

**Authors:** Yang Liu, Konrad Steinestel, Arefeh Rouhi, Milena Armacki, Kristina Diepold, Gabriela Chiosis, Thomas Simmet, Thomas Seufferlein, Ninel Azoitei

**Affiliations:** ^1^ Center for Internal Medicine I, Ulm University, Ulm, Germany; ^2^ Department of Gastroenterology and Hepatology, Zhongda Hospital, Southeast University, Nanjing, China; ^3^ Institute of Pathology and Molecular Pathology, Bundeswehrkrankenhaus Ulm, Ulm, Germany; ^4^ Gerhard-Domagk-Institute of Pathology, University of Münster, Münster, Germany; ^5^ Center for Internal Medicine III, Ulm University, Ulm, Germany; ^6^ Department of Molecular Pharmacology and Chemistry, Memorial Sloan-Kettering Institute, New York, NY, USA; ^7^ Institute of Pharmacology of Natural Products & Clinical Pharmacology, Ulm University, Ulm, Germany

**Keywords:** tumor angiogenesis, hypoxia, VEGF-A, STK33, HIF-1α

## Abstract

Lately, the HSP90 client serine/threonine kinase STK33 emerged to be required by cancer cells for their viability and proliferation. However, its mechanistic contribution to carcinogenesis is not clearly understood. Here we report that elevated STK33 expression correlates with advanced stages of human pancreatic and colorectal carcinomas. Impaired proliferation and augmented apoptosis associated with genetic abrogation of STK33 were paralleled by decreased vascularization in tumor xenografts. In line with this, ectopic STK33 not only promoted tumor growth after pharmacologic inhibition of HSP90 using structurally divergent small molecules currently in clinical development, but also restored blood vessel formation *in vivo*. Mechanistic studies demonstrated that HSP90-stabilized STK33 interacts with and regulates hypoxia-driven accumulation and activation of HIF-1α as well as secretion of VEGF-A in hypoxic cancer cells. In addition, our study reveals a putative cooperation between STK33 and other HSP90 client protein kinases involved in molecular and cellular events through which cancer cells ensure their survival by securing the oxygen and nutrient supply. Altogether, our findings indicate that STK33 interferes with signals from hypoxia and HSP90 to promote tumor angiogenesis and tumor growth.

## INTRODUCTION

STK33 is a serine/threonine kinase that belongs to the Ca^2+^/calmodulin-dependent (CAMK) family of kinases and was revealed by sequencing human chromosome 11 region 11p15 and mouse chromosome 7 [1]. STK33 is highly expressed in a wide range of tissues including fetal lung and heart, testis, retina and brain [2]. Several studies during the last decade revealed the emerging role of STK33 in cancer. The kinase promotes cell migration and invasion while it suppresses p53 gene expression in human large cell lung cancer [3]. Overexpression of STK33 induced liver tumor burst while the knockout suppressed diethylnitrosamine (DEN)-induced mouse liver tumorigenesis [4]. In this study, Yang and colleagues demonstrated a direct binding of STK33 to c-Myc oncogene, an event that resulted in increased c-Myc transcriptional activity. Elevated endogenous STK33 level in hypopharyngeal squamous cell carcinoma (HSCC) was associated with clinico-pathological hallmarks of tumor aggressiveness [5]. In detail, IHC score of STK33 was significantly higher in patients at advanced stages (stage II, III and IV) and associated with lymph node metastasis. STK33 was found to be essential for survival of various mutant KRAS-dependent hematopoietic and epithelial cancer cells [6, 7]. Many protein kinases involved in a wide array of signaling pathways including SRC, AKT, ERBB2/HER2, PKC or PKD2 are clients of the Heat Shock Protein 90 (HSP90) [8, 9, 10, 11, 12, 13, 14]. Using a quantitative protein interaction screen, we previously identified STK33 as a novel client of the HSP90 chaperone [7]. HSP90 is a ubiquitously expressed molecular chaperone that facilitates protein folding, regulates quality control and guides protein turnover in an effort to maintain cellular homeostasis [15, 16]. HSP90 is recruited to its clients through interactions with co-chaperones, such as CDC37, which bridge the interaction between HSP90 and protein kinases [17, 18]. The HSP90/CDC37 complex utilizes ATP to promote and stabilize functional conformations of its clients [16, 18]. Pharmacological inhibition of HSP90 by the ansamycin antibiotic 17-(allylamino)-17-demethoxygeldanamycin (17-AAG) or by PU-H71 leads to destabilization and subsequent proteasomal degradation of clients [7, 14, 15, 19, 20]. HSP90 associates directly with HIF-1α [21, 22] and HSP90 inhibitors have been reported to indirectly regulate the oxygen sensor protein [23, 24, 25]. Furthermore, HSP90 inhibitor geldanamycin destabilizes HIF-1α protein, an event that negatively impacts on VEGF mRNA levels and VEGF transcriptional activity [24]. Since VEGF is one of the most potent mediators of the formation of blood vessels both under physiologic and pathologic conditions, regulation of HIF-1α/VEGF through HSP90 suggests the implication of the chaperone in the vascularization of hypoxic tumors, most likely by stabilization of client proteins. Our previous study showed that pharmacologic inhibition of HSP90 resulted in destabilization and subsequent proteasome-mediated degradation of STK33 in various cancer cell lines. Furthermore, degradation of STK33 by HSP90 inhibition preceded apoptosis while ectopic expression of the kinase was able to rescue cancer cell viability after HSP90 inhibitor treatment [7]. However, it remained unclear whether chaperone-stabilized STK33 participates in tumor progression by affecting cancer cell viability only or whether it is also involved in an HSP90-maintained angiogenic program.

This work reveals a new role of STK33 during tumor progression, namely the participation to the regulation of HIF-1α and its target gene VEGF-A in hypoxic cancer cells. The data indicates the requirement of STK33 in the accumulation of HIF-1α and secretion of VEGF supported by the chaperone. In addition, it raises the hypothesis of potential cooperation between STK33 and other HSP90 client kinase proteins in order to ensure a robust and lasting molecular signaling response during tumor development. Finally, our study favors the use of HSP90 inhibitor PU-H71 (currently undergoing clinical evaluation) to target cancer growth and vascularization particularly in hypoxic tumors with high expression of STK33.

## RESULTS

### STK33 is expressed in human pancreatic and colorectal tumor specimens

**Figure 1:**
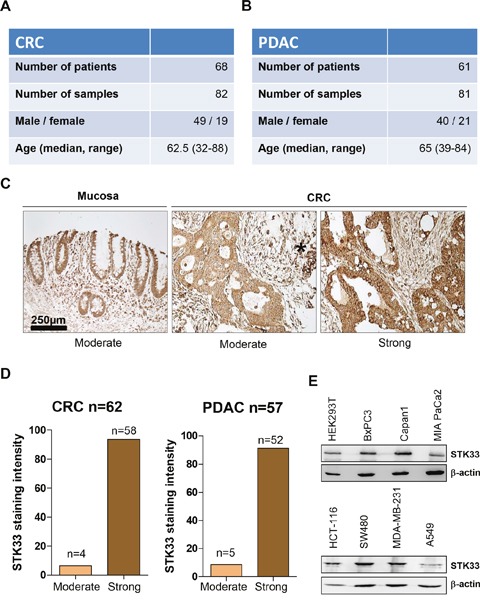
STK33 is expressed in human colorectal (CRC) and pancreatic (PDAC) tumor specimens **(A, B)** patient data including gender and age taken into the study are presented. **(C)** STK33 immunoreactivity was analyzed using standardized protocols. All cancer samples exhibited “moderate” to “strong” STK33 staining intensity and therefore grouped accordingly. Representative set of CRC is depicted. Asterisk indicates immune cells with strong STK33 immunoreactivity. **(D)** percentage of colorectal (CRC) and pancreatic (PDAC) tumor specimens classified according to STK33 staining intensity in “moderate” and “strong” is shown. **(E)** lysates of various cancer cell lines (pancreatic – BxPC3, Capan1, MIA PaCa2; colon – HCT-116, SW480; breast – MDA-MB-231 and lung – A549) were subjected to western blot analysis. Membranes were incubated with STK33-specific antibody. β-actin was used as quality control.

STK33 is expressed in various tissues [2]. In order to interrogate the levels of STK33 in tumor specimens, our study involved a cohort of 68 patients with colorectal cancer (CRC) as well as a set of 61 patients with pancreatic ductal adenocarcinoma (PDAC) (Figure 1A and 1B). 56.71% of CRC and 62.90% of PDAC were classified as grade 2 (G2) while 43.28% of CRC and 37.09% PDAC were grade 3 (G3) respectively (Supplementary Figure 1A and Supplementary Figure 1B). No well-differentiated (G1) tumors were observed in either CRC or PDAC cohorts. Expression of STK33 could be assessed in 62 CRC and 57 PDAC specimens. Virtually all CRC specimens stained positive for STK33 with 93.54% of tumors showing strong and 6.45% of tumors showing moderate STK33 immunoreactivity (Figures 1C, 1D). Similarly, 91.2% of PDAC samples displayed strong and only 8.8% revealed moderate STK33 expression (Figure 1D and Supplementary Figure 1C). Several tumor cell lines including pancreatic, colorectal, breast and lung cancer also express STK33 (Figure 1E). Altogether, these data suggest that STK33 is expressed in tumor tissues and may play an important role in cancer progression.

### Abrogation of STK33 is associated with impaired cancer cell proliferation and enhanced apoptosis

**Figure 2:**
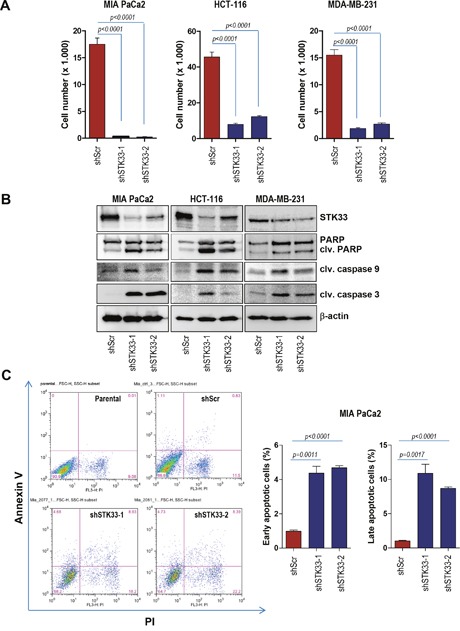
STK33 abrogation is associated with impaired proliferation and augmented apoptosis in tumor cells **(A)** 3 × 10^4^ HCT-116 colon, MIA PaCa2 pancreatic or MDA-MB-231 breast cancer cells transiently transduced with two STK33-specific shRNAs (shSTK33-1 and shSTK33-2) or a non-targeting shRNA (shScr) were plated in 12 well dishes and allowed to grow for the next 72 hours. After 3 days cell number was determined. Error bars represent mean +/- SEM of two independent experiments performed in triplicate. **(B)** protein lysates of stably transduced MIA PaCa2, HCT-116 and MDA-MB-231 cells were subjected to western blot analysis with cleaved PARP, cleaved caspase 3, cleaved caspase 9 and STK33 antibodies. β-actin was used as loading control. **(C)** MIA PaCa2 pancreatic cancer cells stably transduced with STK33-specific (shSTK33-1 and shSTK33-2) or non-coding (shScr) shRNAs were subjected to Annexin V / Propidium Iodide (PI) staining and subsequent flow cytometry. The analysis was conducted in triplicate. Data is shown as mean +/- SEM. One of the two experiments is presented.

We previously identified STK33 as a client of HSP90 [7], a chaperone known to stabilize and activate multiple proteins, several of which are mutated or highly expressed in human cancers [26, 27, 28]. Moreover, our previous findings indicated that STK33 participates to the tumor growth promoted by the chaperone [7]. In order to investigate a potential direct role of STK33 in tumor cell proliferation, several cancer cell lines were stably transduced with lentiviral vectors encoding two STK33-specific shRNAs or a non-coding shRNA. Abrogation of STK33 was associated with impaired proliferation in MIA PaCa2 pancreas, HCT-116 colon and MDA-MB-231 breast cancer cells (Figure 2A, Supplementary Figure 2A). Furthermore, depletion of STK33 highly correlated with increased cell death as revealed by augmented cleaved PARP and cleaved caspase 3 in western blot analysis (Figure 2B). The enhanced cleaved caspase 9 indicates apoptosis induction via mitochondrial pathway (Figure 2B). These results were substantiated by the determination of apoptosis by Annexin V and Propidium Iodide (PI) staining in MIA PaCa2 pancreatic cancer cells (Figure 2C). Here again, STK33 knock-down correlated with increased cell death.

### STK33 deletion is associated with impaired tumor growth and angiogenesis *in vivo*

**Figure 3:**
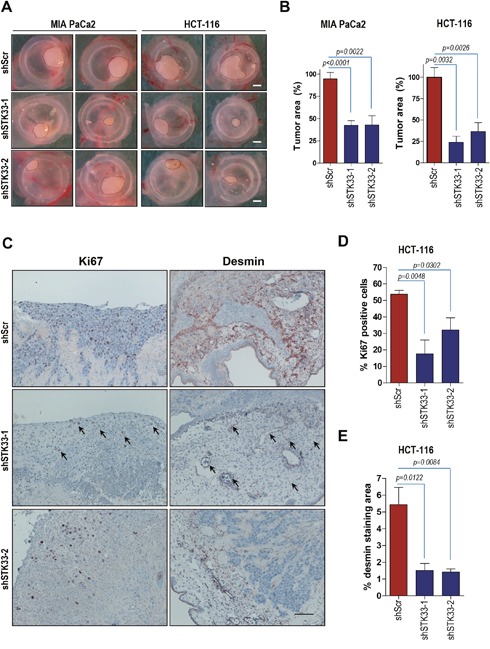
Deletion of STK33 results in decreased tumor growth and impaired tumor-driven vascularization *in vivo* **(A)** 1.5×10^6^ MIA PaCa2 (pancreatic) or HCT-116 (colon) cancer cells transduced with STK33 specific (shSTK33-1 and shSTK33-2) or non-coding (shScr) shRNAs were implanted on chicken CAM on day 8 after fertilization. Tumor formation was monitored for the next 120 hours. Scale bar, 1mm. **(B)** quantification of tumor area is shown. Error bars represent mean +/- SEM of five to eight tumors. **(C)** immunohistochemistry analysis of HCT-116 tumors growing on CAM using specific antibodies directed against Ki67 and desmin is presented. Bar, 125 μm. **(D)** quantification of Ki67 positive HCT-116 cells is shown. Error bars represent mean +/- SEM of at least six microscopic fields, each containing at least 200 cells. **(E)** desmin immunoreactivity in HCT-116 tumors was quantified by subtracting background staining from specific signal using ImageJ software.

Having demonstrated that STK33 is required for cancer cell growth *in vitro*, we further investigated the effect of STK33 abrogation in tumor formation *in vivo* using the chicken chorionallantoic membrane (CAM) assay, an established model of *in vivo* tumor formation [29, 30]. MIA PaCa2 pancreatic and HCT-116 colon cancer cells were transduced with either scrambled oligonucleotide or STK33-specific shRNAs and seeded on the surface of CAM, eight days after egg fertilization. Four days after implantation, tumors were retrieved, photographed, fixed and subjected to immunohistochemistry analysis. Abrogation of STK33 correlated with a significant decrease of tumor size when compared to control tumors (Figure 3A and 3B). Consequently, there was a substantial reduction of the proliferation index, as evidenced by the number of Ki67 positive tumor cells (Figure 3C and 3D). Pancreatic and colon tumors xenografted on chicken CAM are associated with an intense *de novo* vascularization, both in the inner core and in the surrounding CAM tissue [30]. Interestingly, examination of tumor-driven vascularization in xenografts revealed a marked reduction of blood vessel density, as determined by reduced immunoreactivity of desmin (Figure 3C and 3E) and von Willebrand Factor VIII (vWF) (data not shown).

### STK33 mediates hypoxia-induced HIF-1α stabilization and VEGF secretion

**Figure 4:**
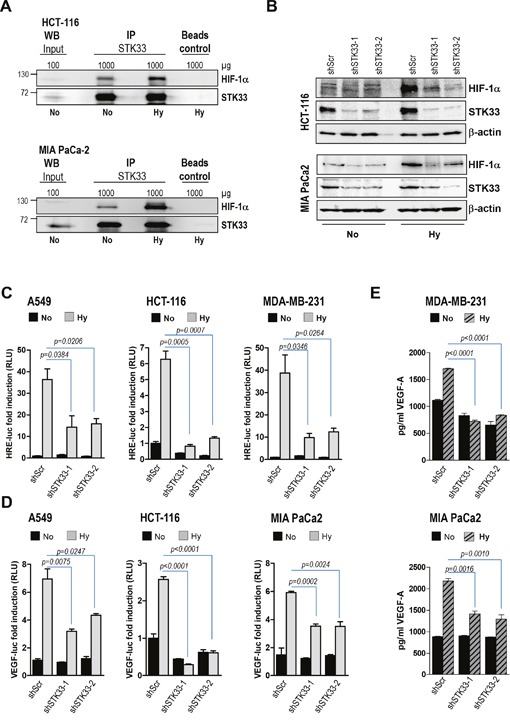
STK33 regulates hypoxia-induced HIF-1α accumulation and VEGF-A secretion **(A)** immunoprecipitation of STK33 was performed with lysates of HCT-116 colon and MIA PaCa-2 pancreatic cancer cells cultivated in low oxygen (Hy) or normal atmosphere (No) conditions. Membranes were re-incubated with STK33 antibody. **(B)** cancer cells stably transduced with non-coding shRNA (shScr) or STK33-specific shRNAs (shSTK33-1 and shSTK33-2) were incubated in normoxic (No) or low oxygen conditions (Hy). HIF-1α abundance was determined using western blot analysis. Membranes were reincubated with STK33-specific antibody to confirm the knock-down and β-actin as loading control. One of three independent experiments is shown for each cell line. **(C)** cancer cells with STK33 deletion were transiently transfected with 3xHRE-luc and pTK-Renilla. Three hours later cells were incubated under normoxic (No) or hypoxic (Hy) conditions as indicated. Luciferase was measured after 24 hours. Bars are the means +/- SEM of at least two independent experiments conducted in triplicate. **(D)** cancer cells were transfected with VEGF-A-luc reporter and pTK-Renilla before incubation in normoxia or low oxygen. Cleared lysates were subjected to promoter assay. Bars indicate the means +/- SEM of at least two independent experiments performed in triplicate. **(E)** supernatants of cancer cells with STK33 knock-down incubated in low oxygen were used for VEGF-A-specific ELISA. Bars represent the means +/- SEM of a representative experiment out of two conducted in triplicate.

Our *in vivo* data imply that STK33 might play an important role in tumor-driven vascularization. Formation of the blood vessels both under physiologic and pathologic conditions involves vascular endothelial growth factor A (VEGF-A) of which upregulation occurs mainly via stabilization of hypoxia-inducing factor 1α (HIF-1α). Consequently, we sought to find out whether STK33 might regulate this oxygen sensor protein in hypoxic cancer cells. Low oxygen atmosphere was achieved by using a dedicated hypoxia kit. Western blot analysis confirmed the stabilization of HIF-1α in various cancer cell lines exposed for eight hours to low oxygen conditions (Supplementary Figure 2B). In our experimental setup STK33 interacted with hypoxia-stabilized HIF-1α in HCT-116 colon, MIA PaCa2 (Figure 4A) and MDA-MB-231 breast cancer cells (Supplementary Figure 2C) suggesting a potential role of STK33 in hypoxic tumors. In order to demonstrate a putative regulation of HIF-1α by STK33, accumulation of the oxygen sensor protein was investigated. Our findings demonstrate that deletion of STK33 markedly reduced hypoxia-induced accumulation of HIF-1α in colon and pancreatic cancer cells (Figure 4B). Decreased HIF-1α abundance in cancer cells with STK33 deletion and exposed to hypoxia was associated with significantly impaired transcriptional activation of the HIF-Responsive Element (HRE), a HIF-1α docking site present in promoters that contain the RCGTG sequence (Figure 4C). Investigation of the HIF-1α - target gene VEGF demonstrated that impaired HIF-1α accumulation following STK33 knock-down correlated with substantially reduced VEGF promoter activity (Figure 4D) and decreased levels of extracellular VEGF (Figure 4E and Supplementary Figure 2D).

### Ectopic STK33 restores tumor-driven vascularization after HSP90 pharmacologic inhibition

**Figure 5:**
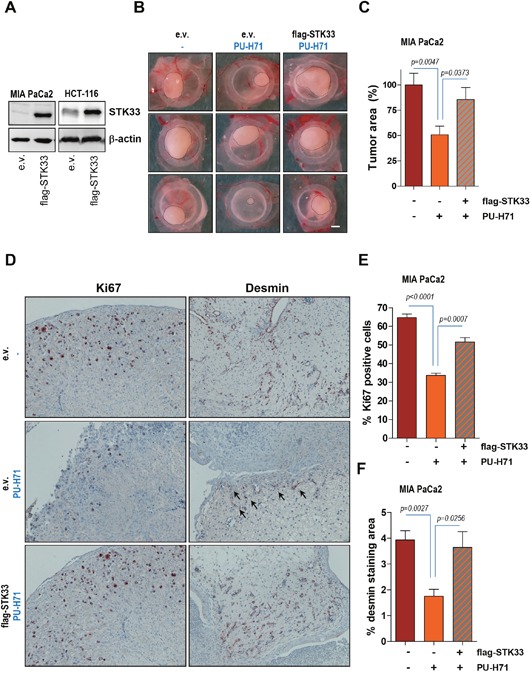
Ectopic STK33 restores the *in vivo* tumor growth and tumor-driven vascularization after HSP90 inhibition **(A)** lysates of cancer cells stably overexpressing STK33 were subjected to SDS-PAGE. Membranes were incubated with STK33 antibodies. β-actin was used as loading control. **(B)** 1.5×10^6^ MIA PaCa2 pancreatic cancer cells stably transduced with STK33 were xenografted on chicken CAM on day 8 after fertilization. Tumor formation was monitored for the next 120 hours. Representative photographs are presented. Scale bar, 1mm. **(C)** quantification of tumor area is shown. Error bars represent mean +/- SEM of five to eight tumors. **(D)** immunohistochemistry analysis of MIA PaCa2 tumors growing on CAM using specific antibodies directed against Ki67 and desmin is presented. **(E)** quantification of Ki67 positive cells is shown. Error bars represent mean +/- SEM of at least six microscopic fields, each containing at least 200 cells. **(F)** desmin immunoreactivity in MIA PaCa2 tumors was quantified by substracting background staining from specific signal using ImageJ software.

Data presented above suggest that STK33 promotes VEGF secretion by interacting with and regulating hypoxia-induced HIF-1α accumulation. Notably, both HIF-1α and STK33 are stabilized by HSP90 chaperone [7, 22]. Given that HSP90 inhibition impairs tumor growth in an STK33-dependent manner [7] we sought to investigate whether STK33 does also participate in *de novo* tumor vascularization promoted by the chaperone. We reconfirmed our previous data showing that abrogation of STK33 following incubation of cancer cells with PU-H71, an ATP-competitive HSP90 inhibitor currently under investigation in clinical trials [31, 32], results in augmented cell death as demonstrated by increased levels of cleaved PARP in western blot analysis (Supplementary Figure 3A). Cancer cells transduced with an expression vector encoding STK33 (Figure 5A) were xenografted on chicken CAM. Treatment of tumors with 1 μM PU-H71 resulted in a significant decrease in tumor size (Figure 5B and 5C). However, overexpression of STK33 partially reverted the cytotoxicity of PU-H71 as indicated by restored tumor formation (Figure 5B and 5C) and was associated with higher number of Ki67 positive cancer cells as compared with tumors transduced with empty vector (Figure 5D and 5E). Most importantly, enhanced proliferation in tumors ectopically expressing STK33 was paralleled by augmented levels of desmin (Figure 5D and 5F), an observation that suggests a causal relationship between STK33 expression and tumor angiogenesis downstream of HSP90.

### STK33 contributes to the tumor angiogenesis program orchestrated by HSP90

**Figure 6:**
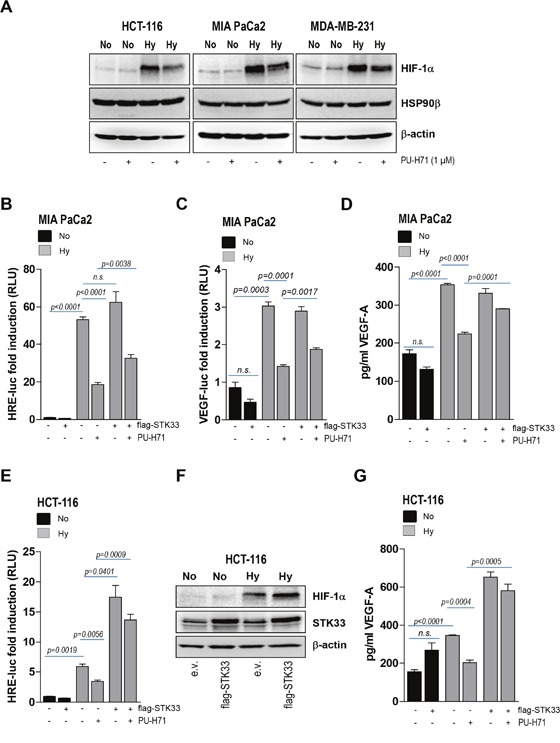
Ectopic STK33 rescues HIF-1α stabilization and VEGF secretion after HSP90 inhibition **(A)** lysates of various cancer cells pre-treated for 12 hours with 1 μM PU-H71 and further incubated for the next 8 hours under hypoxic conditions were prepared. Western blot analysis was conducted with HIF-1α and HSP90β-specific antibodies. β-actin was used as loading control. **(B, C)** MIA PaCa2 cancer cells stably overexpressing STK33 (flag-STK33) were transiently transfected either with 3xHRE-luc (B) or VEGF-luc (C) and pTK-Renilla. Next day cells were incubated in low oxygen and/or 1 μM PU-H71 as indicated. Luciferase was measured after 24 hours. Bars are the means +/- SEM of at least two independent experiments conducted in triplicate. **(D)** supernatants of cancer cells stably transduced with STK33 or empty vector and incubated in low oxygen were used for VEGF-A-specific ELISA. Bars represent the means +/- SEM of a representative experiment out of two conducted in triplicate. **(E)** HCT-116 cancer cells stably overexpressing STK33 (flag-STK33) were transiently transfected with 3xHRE-luc and pTK-Renilla. Next day cells were incubated under normoxic (No) or hypoxic (Hy) conditions as indicated. Luciferase was measured 24 hours later. Bars are the means +/- SEM of at least two independent experiments conducted in triplicate. **(F)** cancer cells stably overexpressing STK33 (flag-STK33) were incubated in low oxygen atmosphere. Cleared cell lysates were used for western blot analysis with HIF-1α antibody. Membranes were reprobed with STK33 and β-actin. **(G)** supernatants of HCT-116 cells stably transduced with STK33 or empty vector, incubated in low oxygen atmosphere and/or 1 μM PU-H71 for 24 hours were used for VEGF-A-specific ELISA. Bars represent the means +/- SEM of a representative experiment out of two conducted in triplicate.

The experiments presented in Figure 4 demonstrate that STK33 is involved in the regulation of HIF-1α accumulation and activation as well as subsequent VEGF secretion in hypoxic tumors. Interestingly, the bHLH-PAS domain of HIF-1α was shown to interact with HSP90 while its activation in hypoxia was inhibited in the presence of geldanamycin [22]. This data is in line with our findings showing that pharmacologic inhibition of the chaperone (Figure 6A) or deletion of HSP90β (Supplementary Figure 3B) resulted in decreased hypoxia-induced HIF-1α accumulation. Next, we interrogated the potential involvement of STK33 in HIF-1α regulation by HSP90 in hypoxic tumor cells. Promoter analysis using a HIF-specific reporter revealed that overexpression of STK33 was able to partially restore HIF-1α transcriptional activity in MIA PaCa2 pancreatic cancer cells treated with PU-H71 and incubated in hypoxic atmosphere (Figure 6B). Furthermore, ectopic STK33 also partially rescued VEGF promoter activity (Figure 6C) as well as VEGF secretion (Figure 6D and Supplementary Figure 4A) in hypoxic cancer cells subjected to HSP90 inhibition. Surprisingly, overexpression of STK33 in hypoxic HCT-116 colon cancer significantly boosted HIF-1α promoter activity beyond the levels achieved by hypoxic cells transduced with empty vector (Figure 6E, lane 5 vs. lane 3). This was corroborated also with a further accumulation of HIF-1α (Figure 6F), an effect not observed in other cancer cell lines taken into the study (Supplementary Figure 4B). In line with these observations, overexpression of STK33 in hypoxic HCT-116 resulted in a further increase of VEGF secretion as compared to empty vector transduced cells incubated in low oxygen (Figure 6G, lane 5 vs. lane 3). Altogether, these findings suggest that STK33 participates in blood vessel formation promoted by HSP90 chaperone by regulating HIF-1α. They also indicate that STK33 displays a functional versatility that translates into different magnitudes of response in different cell lines / tumor types.

### STK33 putatively cooperates with other HSP90 client proteins

**Figure 7:**
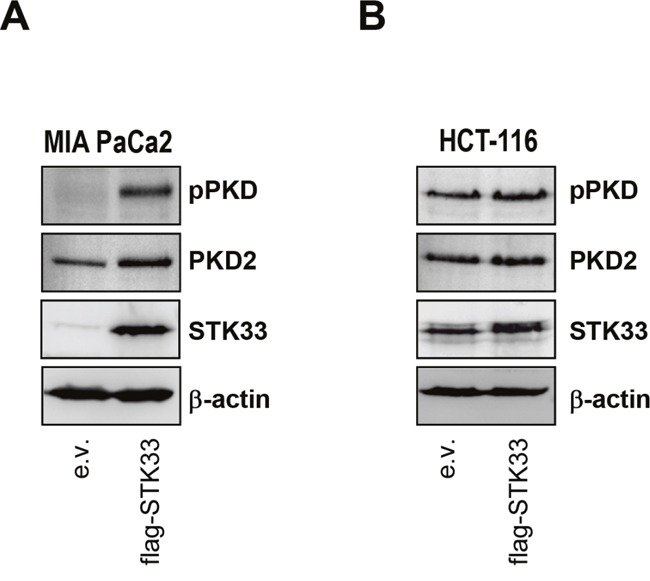
STK33 cooperates with other protein kinase clients stabilized by HSP90 during tumor progression **(A, B)** lysates of MIA PaCa2 pancreas (A) and HCT-116 colon (B) cancer cells stably overexpressing STK33 (flag-STK33) were subjected to SDS-PAGE analysis with pPKD and PKD2 antibodies. Membranes were reprobed with STK33 and β-actin.

In tumor cells HSP90 aids in folding a plethora of “client” proteins and helps to sustain their aberrant activity. The data above demonstrate that one HSP90 client protein (STK33, a kinase) is able to regulate another client (HIF-1α, a transcription factor) in hypoxic tumors. We were reminded of an earlier work showing that serine/threonine kinase PKD2 not only regulates HIF-1α, but also interacts with and participates to various functions coordinated by HSP90, including tumor growth and angiogenesis [14]. We therefore asked whether STK33 could activate PKD2. When investigating such a possibility we found that overexpression of STK33 was paralleled by an augmented PKD2 expression and activity particularly in MIA PaCa2 pancreatic cancer cells as demonstrated by the western blot analysis (Figure 7A). While this observation could not be reproduced in other cancer cell lines (Figure 7B) it raises the possibility that, at least in some tumor entities, more than one HSP90 client protein is involved in order to achieve a full and fast response to a certain cellular condition.

## DISCUSSION

High proliferation and an improper vascular architecture lead to an intratumoral hypoxic environment in solid tumors. The acidic microenvironment around hypoxic tumor cells is accompanied by the activation of a myriad of protein kinases, many of which are stabilized by HSP90. Recently we identified STK33 as a client of HSP90 and demonstrated that is required for cancer cell viability supported by the chaperone. However, the exact molecular mechanism of how STK33 is involved in tumor progression remained elusive to date.

**Figure 8:**
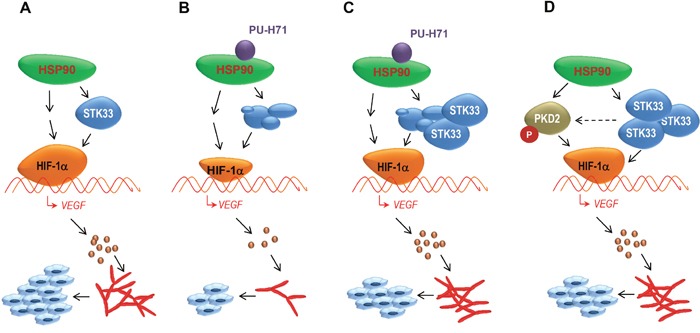
STK33 contributes to tumor growth and angiogenesis orchestrated by HSP90 chaperone by regulating HIF-1α / VEGF signaling pathway **(A)** among other client proteins stabilized by HSP90 chaperone, STK33 is implicated in tumor growth and angiogenesis. **(B)** degradation of STK33 following pharmacologic inhibition of HSP90 negatively impacts on HIF-1α accumulation and VEGF secretion in hypoxic tumors. Consequently, tumor growth and tumor-driven vascularization are significantly suppressed. **(C)** ectopic STK33 partially restores HIF-1α accumulation and VEGF release in hypoxic cancer cells subjected to pharmacologic HSP90 inhibition. **(D)** high expression of STK33 is associated (at least in some tumor entities) with augmented PKD2 phosphorylation, another HSP90 client kinase involved in tumor blood vessel formation. Thus, HSP90 chaperone may promote vascularization of tumors by coordinating (at least) two client proteins aiming at a fast & robust activation of HIF-1α/VEGF signaling pathway.

In this work we identified STK33 as an essential HSP90 client that participates in a complex tumor angiogenic program coordinated by the chaperone. Our study has revealed STK33 as an essential molecule for progression of pancreatic and colorectal cancers in which the tumor environment was reported to be highly hypoxic [33, 34]. Accordingly, we found a predominantly strong STK33 expression in all investigated human CRCs and PDACs. STK33 knock-down resulted in impaired cancer cell proliferation and increased apoptosis. The *in vitro* findings were corroborated with data from tumor xenograft experiments showing a close association between decreased proliferation index and impaired tumor-driven vasculature *in vivo*. To the best of our knowledge, these data are the first to reveal a potential role of STK33 in angiogenesis. The fact that STK33 is a client of HSP90, a chaperone previously shown to be involved in the regulation of HIF-1α [22, 23, 24], prompted us to explore whether STK33 might “serve” HSP90 by affecting the expression and the activity of this oxygen sensor protein. Firstly, we found that endogenous STK33 interacted with hypoxia-stabilized HIF-1α; secondly, deletion of STK33 in tumor cells subjected to low oxygen atmosphere resulted in a reduced HIF-1α accumulation which thirdly, was associated with impaired hypoxia-induced HIF-1α promoter activity. Since VEGF-A is secreted by tumor cells through the activation of HIF-1α [35] we reasoned that STK33 might affect VEGF levels. Indeed, impaired STK33-dependent HIF-1α expression was paralleled by decreased VEGF transcriptional activity and secretion. These results were corroborated with our *in vivo* data depicting not only smaller tumors with decreased Ki67 immunoreactivity but also with low signal of desmin immunostaining suggesting a poor vascularization of tumors with abrogated STK33. In line with several reports, reduced expression or impaired activity of HSP90 was associated with impaired HIF-1α accumulation [23, 24]. The next obvious question was whether STK33 participates in the molecular signaling cascades orchestrated by HSP90. In our experimental model, cancer cells overexpressing STK33 and exposed to HSP90 inhibitor displayed a partial rescue of HIF-1α transcriptional activity and secreted VEGF-A. In line with these results, STK33 overexpression restored vascularization *in vivo* in tumors treated with PU-H71. These findings demonstrate an essential role played by STK33 in another cancer hallmark supported by HSP90, namely tumor angiogenesis (Figure 8). Furthermore, to our surprise, we found that at least in certain cancer cell lines / tumor entities, elevated STK33 expression can further boost the hypoxia-triggered HIF-1α activation and subsequent vascularization. These data may suggest a synergistic effect between high STK33 levels and hypoxic environment toward an accelerated cancer progression. In other words, tumors highly expressing STK33 are prone to a rapid development once they reach a certain level of hypoxia, a fact that would render the expression of STK33 as tumor marker for hypoxic cancers. Given that HSP90 stabilizes multiple proteins implicated in cell viability and proliferation [36], it often remained unclear whether cell killing upon inhibition of HSP90 was a result of depletion of a single client, a simultaneous degradation of multiple proteins or, as we newly hypothesize, a potential cooperation between several HSP90 client protein kinases. In an attempt to address this issue we found that STK33 overexpression in MIA PaCa2 pancreatic cancer cells was associated with augmented kinase activity and expression of PKD2 (Figure 8), another serine/threonine kinase described as a client of HSP90 [14]. Since PKD2 also regulates HIF-1α [14] it is easy to conceive that these two HSP90 client proteins could simultaneously or successively participate to HIF-1α—dependent VEGF release in order to ensure a fast and robust angiogenic response. This first observation opens new avenues and urges for further investigation of STK33 – PKD2 putative reciprocal phosphorylation and functional compensation, potential physical interaction and potential presence in the same complex with their master chaperone.

In conclusion, our study 1) demonstrates a new function for STK33 in several *in vitro* and *in vivo* experiments, namely tumor angiogenesis downstream of HSP90 chaperone while it 2) characterizes the role of the kinase in the activation of the HIF-1α transcription factor and its target gene, VEGF, in hypoxic cancers. Furthermore, it 3) raises the hypothesis of potential cooperation between various HSP90 client kinase proteins such as STK33 and PKD2, for a more robust and lasting effect during tumor development. Lastly, our study may have clinical implications since several HSP90 inhibitors are currently being developed as anti-cancer drugs.

## MATERIALS AND METHODS

### Cell lines

MIA PaCa2 pancreatic, MDA-MB-231 breast, A549 lung cancer cell lines were cultured in DMEM (Invitrogen, Germany) supplemented with 10% fetal calf serum (FCS: PAA, Germany), 1% penicillin/streptomycin. HCT-116 colorectal carcinoma cells were cultured in McCoy medium (Sigma, Germany). In our study we aimed at using cancer cell lines that simultaneously fulfill several criteria such as strong response to hypoxic environment, secretion of VEGF in culture supernatants and capacity to form tumors *in vivo*. Cancer cell lines were obtained from DSMZ-German Collection of Microorganisms and Culture Cells (Braunschweig, Germany) or authenticated at the Multiplexion (Heidelberg, Germany).

### Short hairpins and lentiviral transduction

RNAi experiments were performed using pLKO.1 lentiviral shRNA vectors obtained from the TRC-Hs 1.0 (human) shRNA library through Open Biosystems. Two STK33 hairpins (shSTK33-1, #TRCN0000002077 - CCAAGGAACAGCAACCAAGTA and shSTK33-2, #TRCN0000002081 - CTTGCCATTAACTTGCTGCTA) that showed the highest efficacy knock-down were selected. Knock-down of HSP90β was achieved using an HSP90-specific shRNA (#TRCN0000008748) from Open Biosystems. High-titer virus-containing supernatants of HEK293FT cells after transient co-transfection of lentiviral vectors with pMD2.G and psPAX2 packaging vectors were used for lentiviral mediated transduction of cancer cells. Cells were selected either for 3 days (puromycin, 2 μg/ml) or 5 days (blasticidin, 5 μg/ml), allowed 3 days to recover after selection in growing media and then subjected to different assays.

### qPCR analysis

RNA was extracted from cultured cells with RNeasy® Mini Kit (Qiagen, Germany) according to the manufacturer's instructions. cDNA were prepared from 250 ng of total RNA using the iScript™ cDNA Synthesis Kit (Biorad). Validated qPCR primers were obtained from Qiagen, Germany (# 330001 – PPH12688A-200; class of validation: RT^2^ qPCR primer assay). PCR reactions were performed with iTaq™ Universal SYBR® Green Supermix reaction mixture (Biorad) according to the manufacturer's instructions. The thermal cycling conditions were comprised of 2 min at 95°C, followed by 40 cycles of 15 sec at 95°C denaturation and 1 min at 60°C anneal/extension. Data collection was performed during each extension phase. For each of the RNA extractions, measurements of gene expression were obtained in duplicates, and the mean value was used for further analysis. The relative gene expression levels were calculated by “delta-delta Ct method” (ΔΔCt method).

### Hypoxia experiments

The hypoxic atmosphere was achieved by using Anaerocult A Mini Kit (# 1.01611.0001) from Merck, Germany. Cells were incubated for 8 hours to 24 hours as indicated.

### Co-IP and western blot analysis

Whole cell extracts were prepared using a lysis buffer containing 10 mM Tris-HCl, 5 mM EDTA, 50 mM NaCl, 50 mM NaF and 1% Triton X100 supplemented with Complete Protease Inhibitor Cocktail and PhosStop tablets (Roche). After measuring the protein content via Bradford, lysates were subjected to SDS-PAGE and separated proteins transferred to PVDF membranes (Millipore, Massachusetts, USA). For co-IP, protein extracts (IP - 1 mg, input - 100 μg) were incubated with 3 μl STK33 antibody (0.36 μg/μl) and Protein G-Sepharose (GE Healthcare). For western blot analysis 100 μp protein was used. Membranes were blocked with 5% non-fat dry milk in phosphate buffered saline (PBS) containing 0.2% Tween 20 and incubated over night at 4°C with specific antibodies. For subsequent washes 0.2% Tween 20 in PBS was used. The following antibodies were used: STK33 (Abnova, clone 4F7, #H00065975, dilution 1:1000); HIF-1α (BD Transduction Laboratories, #610959, dilution 1:50); cleaved PARP (Cell Signaling, #9505S, dilution 1:700); cleaved caspase 3 (Cell Signaling, #9661, dilution 1:500); HSP90β, (#clone D-19, Santa Cruz Biotechnology, #sc-1057, dilution 1.500); phospho-PKD/PKCμ - Ser744/Ser748 (Cell Signaling, #2054S, dilution 1:1000) and β-actin (Sigma, #A1978, dilution 1:2000).

### Promoter assays

Cancer cells plated in 12-well dishes were transfected with 330 ng/ml of the respective promoter plasmid as indicated in the figure legends using Lipofectamine 2000 (Invitrogen #15338-100) according to the manufacturer instructions. Luciferase activity was determined using the Dual Luciferase Assay Kit (Promega, Mannheim, Germany). Firefly luciferase units were normalized with Renilla luciferase after co-transfection with 17 ng/ml pRL-TK plasmid (Promega, Germany).

### FACS analysis

Annexin-V / Propidium Iodide (PI) staining was performed via flow cytometry according to the manufacturer's guidelines. Annexin V PE Apoptosis Detection Kit PE was obtained from eBiosciences (#88-8102-72). Cells were selected for 3 days with puromycin after viral transduction and allowed to grow for another 3 days in antibiotics-free cultivation media before FACS analysis was conducted. Flow cytometry was performed using FACSalibur (Becton Dickinson) and the data was analyzed by FlowJo software.

### CAM assay

1.5 × 10^6^ MIA PaCa2 pancreatic or HCT-116 colon cancer cells transduced with shSTK33 or non-coding shRNA were xenografted within a 5 mm silicon ring on the surface of the chorionallantoic membrane (CAM) of chicken eggs 8 days after fertilization. Four days after implantation (day 12 after fertilization) tumors were retrieved, fixed in formalin and further subjected to immunohistochemistry.

### Immunohistochemistry of CAM tumors

Formalin-fixed tumors were embedded in paraffin using standard procedures. The 5 μm sections were processed and stained with antibodies directed against Ki67 (1:100; Dako, clone MIB-1) and desmin (1:80; Dako, clone D33).

### Tissue samples and TMA construction

We used 8 different TMAs (4 PDAC/4 CRC) containing at least two representative cores per case (core diameter 1 mm). Cases were collected from the archive of the Gerhard Domagk Institute of Pathology of the University of Münster. Multiple cores included also non-neoplastic pancreatic ducts or non-neoplastic colonic mucosa as an internal control. Selected areas of interest were confirmed by two experienced pathologists before and after TMA construction. In total, 81 PDAC samples and 82 CRC tumor samples were investigated. The use of human tissue samples was approved by the ethics committee of the University of Münster (Az. 2015-102-f-S and 2015-628-f-S, respectively).

### Immunohistochemistry and assessment of STK33 expression

Immunhistochemistry (IHC) was performed on 4-μm-thick paraffin sections from the TMAs using the peroxidase-conjugated avidin-biotin method with a polyclonal rabbit anti-STK33 antibody (Atlas Antibodies, Sweden, 1:50 dilution). In brief, sections were deparaffinized in xylene and rehydrated through graded ethanol at room temperature. Incubation with the primary antibodies was performed for 30 minutes at room temperature. After washing, the sections were incubated with biotinylated secondary antibodies. Immunoreactions were visualized using a 3-amino-9-ethylcarbazole as a substrate (Ventana Optiview DAB IHC detection KIT, Ref: 760-700, Germany). STK33 expression was evaluated by an experienced pathologist (KS) on immunostained TMA slides. Staining intensity was scored as negative/weak, moderate, or strong.

### Statistics

Analyses were performed using GraphPrism 5.0 software GraphPad software, La Jolla, CA, USA). Statistical significance was assessed by an unpaired Student t test. p<0.05 was considered as significant. IHC quantifications were performed using Image J software (National Institute of Health, Bethesda, MD, USA) using the Colour Deconvolution plugin.

## SUPPLEMENTARY MATERIALS FIGURES


