# Supplementary material for: STK33 participates to HSP90-supported angiogenic program in hypoxic tumors by regulating HIF-1α/VEGF signaling pathway
Source: Oncotarget. 2017 Aug 24;8(44):77474–88. doi: 10.18632/oncotarget.20535 (PMC5652794; doi:10.18632/oncotarget.20535)
Supplement: Supplementary file 1 [file oncotarget-08-77474-s001.pdf]

STK33 participates to HSP90-supported angiogenic program in hypoxic tumors by regulating HIF-1α/VEGF signaling pathway

SUPPLEMENTARY MATERIALS

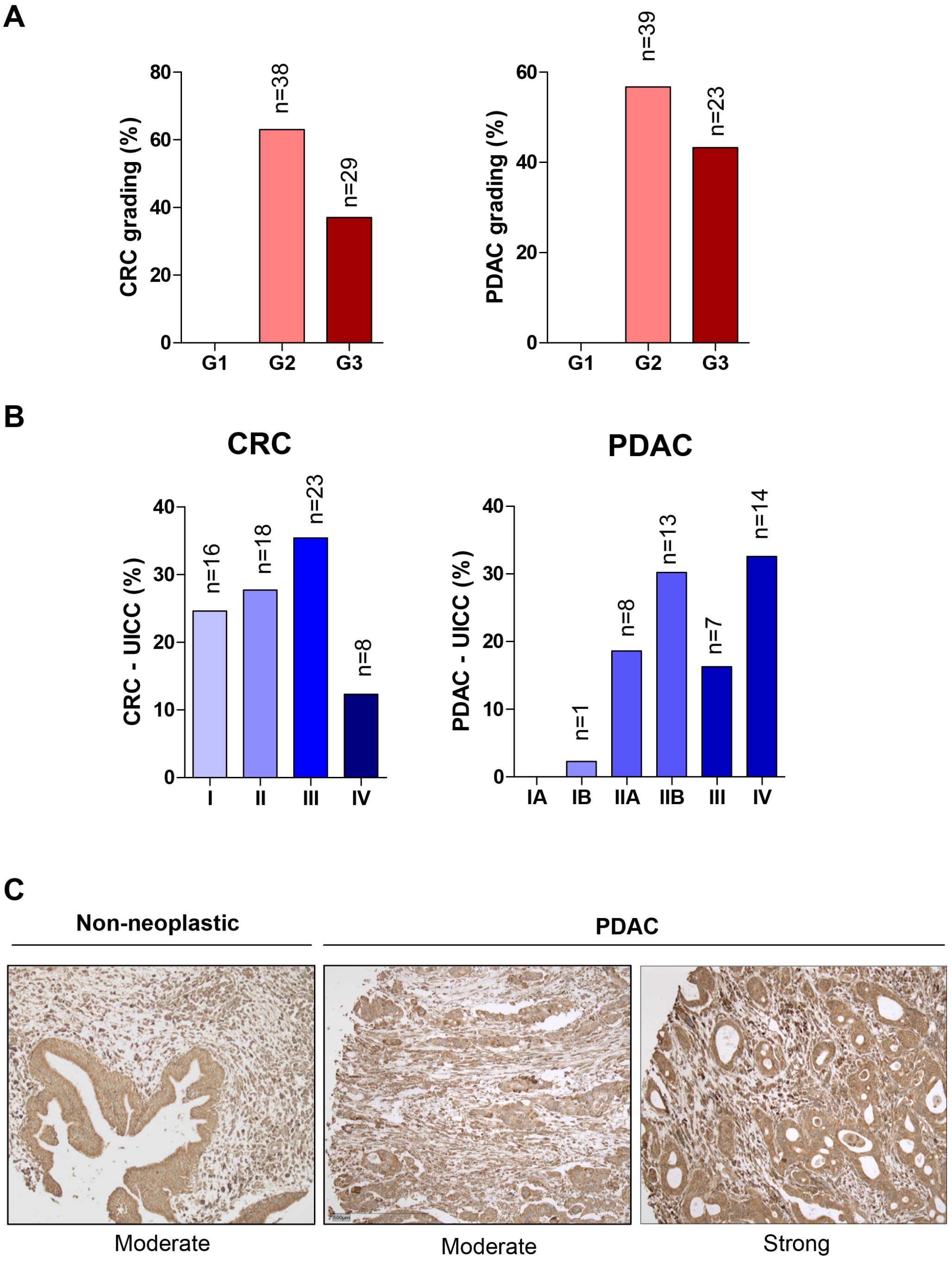

Supplementary Figure 1: STK33 elevated expression correlates with higher tumor grading in human colorectal (CRC) and pancreatic (PDAC) tumor specimens. In order to interrogate the levels of STK33 in tumor specimens, we involved in our study a cohort of 68 patients with colorectal cancer (CRC) as well as a set of 61 patients diagnosed with pancreatic ductal adenocarcinoma (PDAC). Grading **(A)** and UICC **(B)** classification for CRC and PDAC taken into the study are presented. **(C)** STK33 immunoreactivity was analyzed using standardized protocols. All cancer samples exhibited "moderate" to "strong" STK33 staining intensity and therefore grouped accordingly. Representative set of PDAC is depicted.

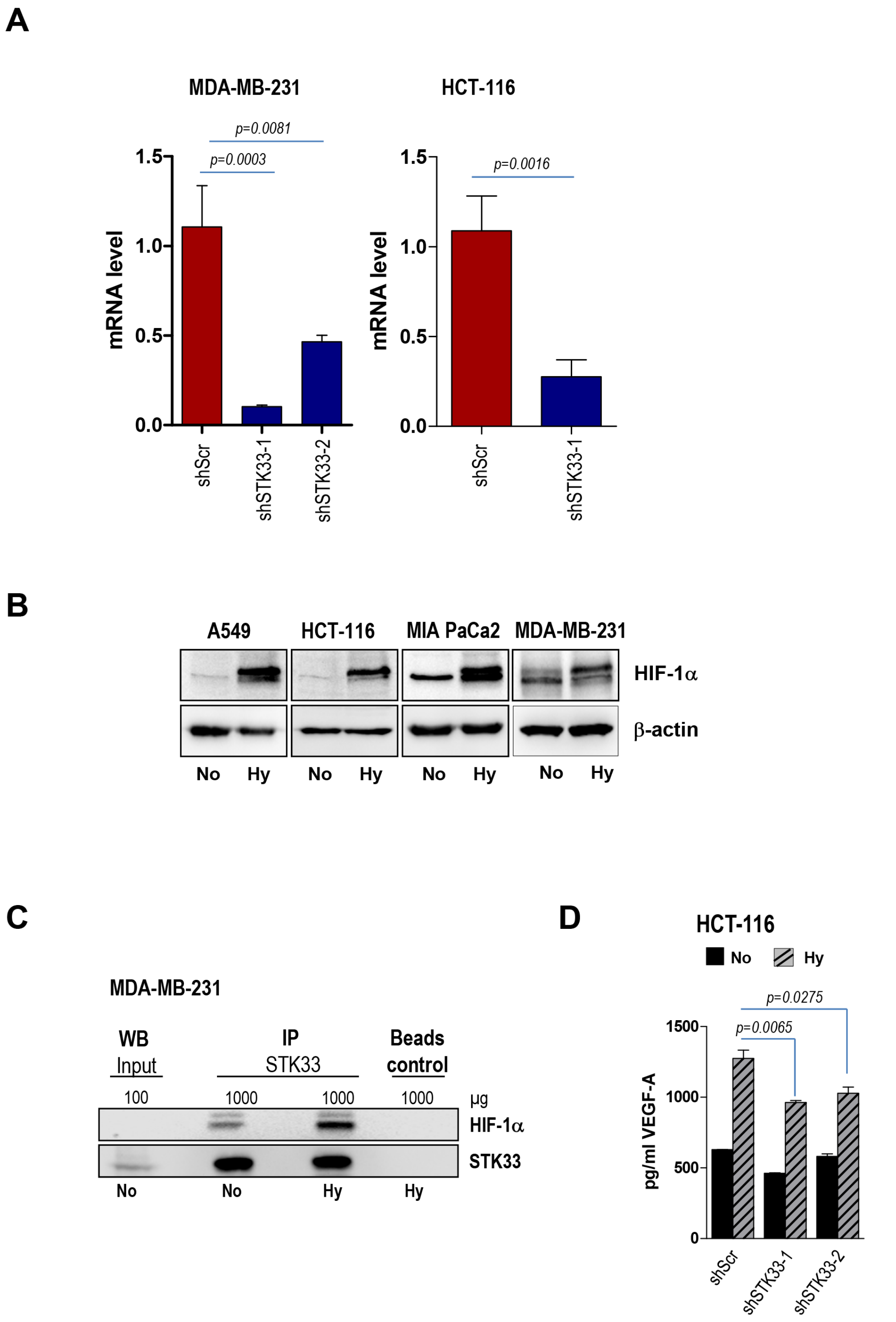

Supplementary Figure 2: STK33 regulates hypoxia-induced VEGF-A secretion. (A) RNA was extracted from cultured cells and subsequent cDNA was prepared. qPCR analysis is presented. For each of the RNA extractions, measurements of gene expression were obtained in duplicates, and the mean value was used for further analysis. The relative gene expression levels were calculated by "delta-delta Ct method" (ΔΔCt method). **(B)** various cancer cell lines were incubated in normoxic (No) or hypoxic (Hy) atmosphere for eight hours. Lysates were subjected to western blot analysis. Membranes were incubated with HIF-1α specific antibody. β-actin was used as loading control. **(C)** immunoprecipitation of endogenous STK33 was performed with lysates of MDA-MB-231 breast cancer cells cultivated in low oxygen or normal atmosphere conditions. Membranes were re-incubated with STK33 antibody. **(D)** supernatants of HCT-116 colon cancer cells with depleted STK33 and incubated in low oxygen were used for VEGF-A-specific ELISA. Bars represent the means +/- SEM of a representative experiment out of two conducted in triplicate.

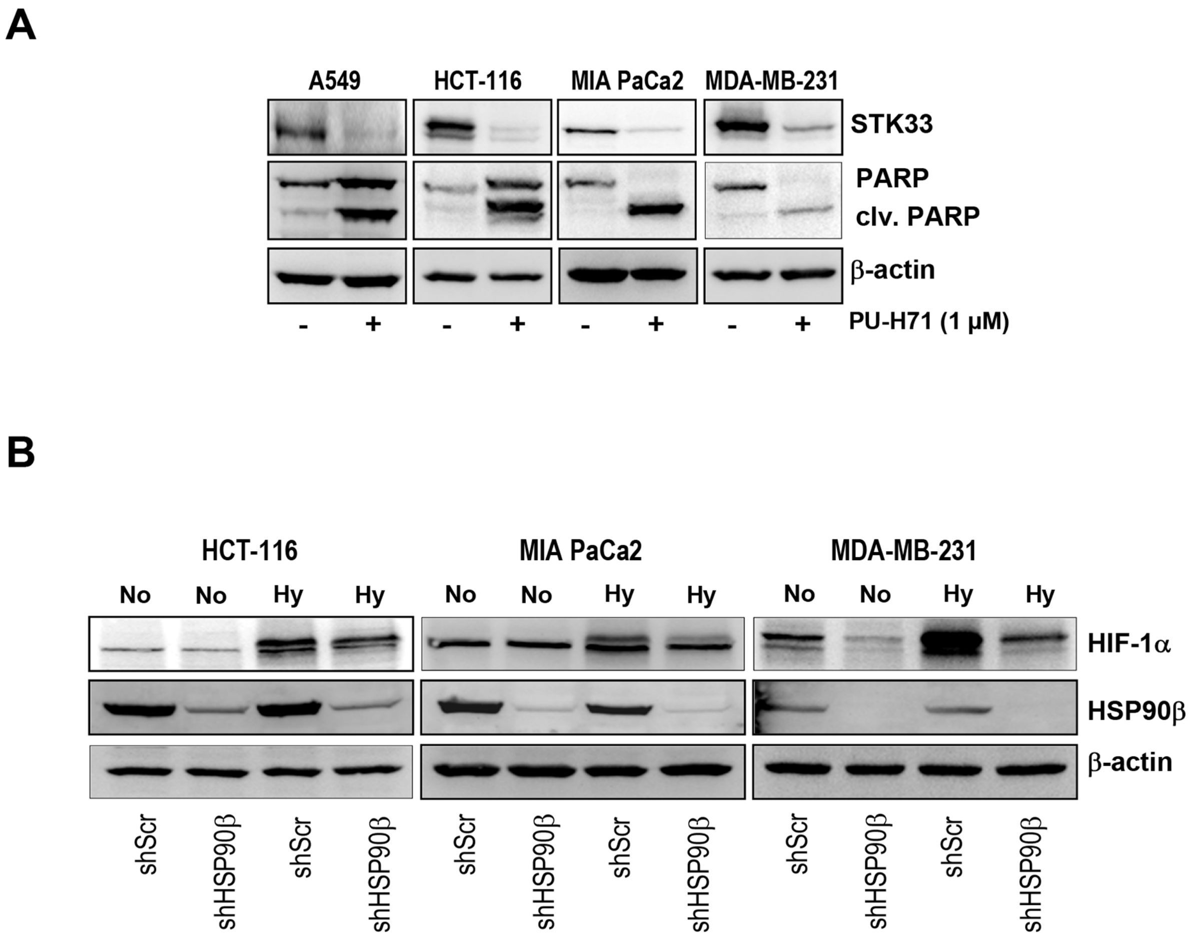

Supplementary Figure 3: HSP90 inhibition-triggered degradation of STK33 is associated with augmented cell death. **(A)** several cancer cell lines were incubated with 1 μM PU-H71 for 24 hours. Cell lysates were prepared and western blot analysis was conducted with cleaved PARP specific antibody. Membranes were incubated with STK33 for confirmation of degradation and β-actin as loading control. **(B)** lysatesof various cancer cells with deleted HSP90β and incubated 8 hours under hypoxic conditions were prepared. Western blot analysis was conducted with HIF-1α and HSP90β-specific antibodies. β-actin was used as loading control.

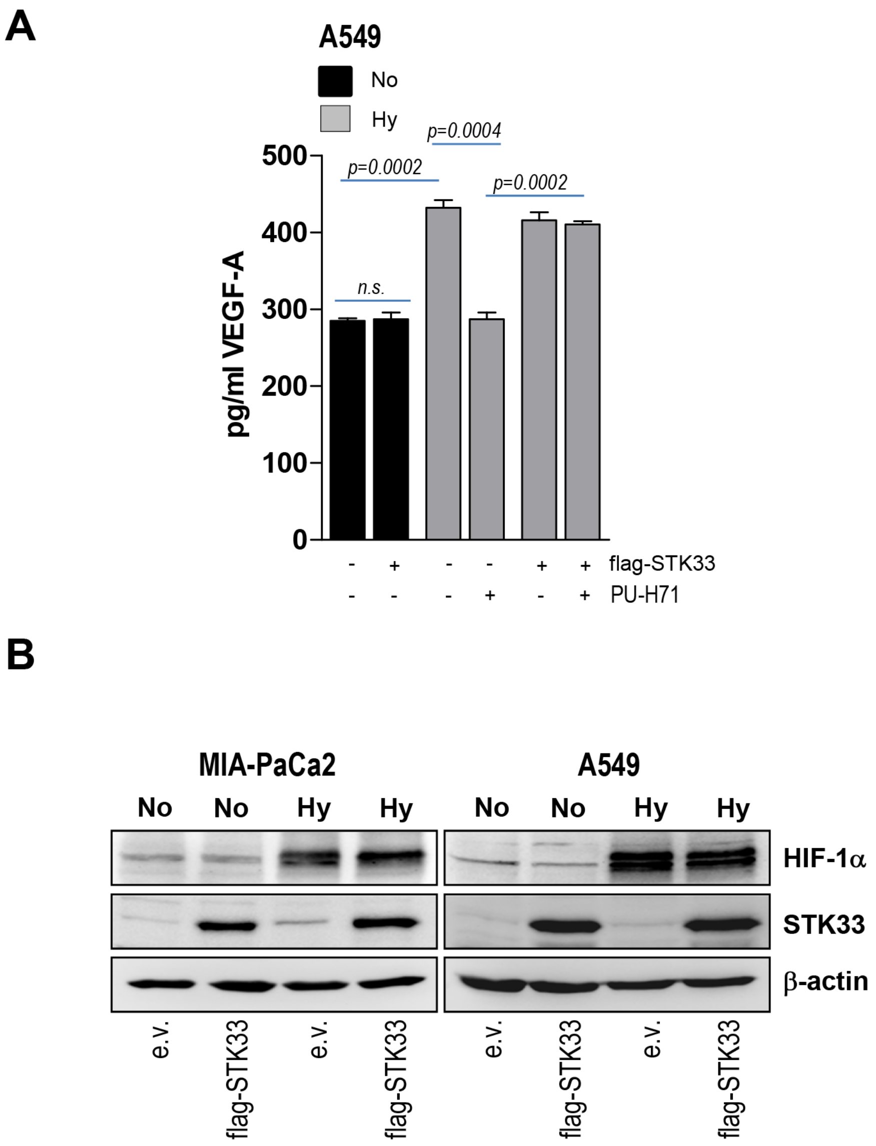

Supplementary Figure 4: Ectopic STK33 restores hypoxia-induced VEGF-A release after HSP90 inhibition. (A) supernatants of A549 lung cancer cells stably transduced with STK33 or empty vector, incubated in low oxygen and/or with 1μM PU-H71 were used for VEGF-A-specific ELISA. Bars show the means +/- SEM of a representative experiment out of two conducted in triplicate. **(B)** MIA PaCa2 and A549 cancer cells stably overexpressing STK33 were incubated in low oxygen atmosphere. Cleared cell lysates were used for western blot analysis with HIF-1α antibodies. Membranes were reprobed with STK33 and β-actin.
